# Population genetic admixture and evolutionary history in the Shandong Peninsula inferred from integrative modern and ancient genomic resources

**DOI:** 10.1186/s12864-024-10514-9

**Published:** 2024-06-18

**Authors:** Haoran Su, Mengge Wang, Xiangping Li, Shuhan Duan, Qiuxia Sun, Yuntao Sun, Zhiyong Wang, Qingxin Yang, Yuguo Huang, Jie Zhong, Jing Chen, Xiucheng Jiang, Jinyue Ma, Ting Yang, Yunhui Liu, Lintao Luo, Yan Liu, Junbao Yang, Gang Chen, Chao Liu, Yan Cai, Guanglin He

**Affiliations:** 1 Genetic and Prenatal Diagnosis Center, Affiliated Hospital of North Sichuan Medical College, Nanchong, 637007 Sichuan China; 2 Institute of Rare Diseases, West China Hospital of Sichuan University, Sichuan University, Chengdu, 610000 China; 3 School of Laboratory Medicine, North Sichuan Medical College, Nanchong, 637007 Sichuan China; 4 Center for Archaeological Science, Sichuan University, Chengdu, 610000 China; 5 Research Center for Genomic Medicine, North Sichuan Medical College, Nanchong, 637100 China; 6 School of Forensic Medicine, Kunming Medical University, Kunming, 650500 China; 7 Institute of Basic Medicine and Forensic Medicine, North Sichuan Medical College and Genetic and Prenatal Diagnosis Center, Affiliated Hospital of North Sichuan Medical College, Nanchong, 637007 Sichuan China; 8 Department of Forensic Medicine, College of Basic Medicine, Chongqing Medical University, Chongqing, 400331 China; 9 West China School of Basic Science & Forensic Medicine, Sichuan University, Chengdu, 610041 China; 10 School of Forensic Medicine, Shanxi Medical University, Jinzhong, 030001 China; 11 Anti-Drug Technology Center of Guangdong Province, Guangzhou, 510230 China; 12 Hunan Key Laboratory of Bioinformatics, School of Computer Science and Engineering, Central South University, Changsha, 410075 China

**Keywords:** Population genetics, Northern Han, Evolution, Genomics, Adaptation

## Abstract

**Background:**

Ancient northern East Asians (ANEA) from the Yellow River region, who pioneered millet cultivation, play a crucial role in understanding the origins of ethnolinguistically diverse populations in modern China and the entire landscape of deep genetic structure and variation discovery in modern East Asians. However, the direct links between ANEA and geographically proximate modern populations, as well as the biological adaptive processes involved, remain poorly understood.

**Results:**

Here, we generated genome-wide SNP data for 264 individuals from geographically different Han populations in Shandong. An integrated genomic resource encompassing both modern and ancient East Asians was compiled to examine fine-scale population admixture scenarios and adaptive traits. The reconstruction of demographic history and hierarchical clustering patterns revealed that individuals from the Shandong Peninsula share a close genetic affinity with ANEA, indicating long-term genetic continuity and mobility in the lower Yellow River basin since the early Neolithic period. Biological adaptive signatures, including those related to immune and metabolic pathways, were identified through analyses of haplotype homozygosity and allele frequency spectra. These signatures are linked to complex traits such as height and body mass index, which may be associated with adaptations to cold environments, dietary practices, and pathogen exposure. Additionally, allele frequency trajectories over time and a haplotype network of two highly differentiated genes, *ABCC11* and *SLC10A1*, were delineated. These genes, which are associated with axillary odor and bilirubin metabolism, respectively, illustrate how local adaptations can influence the diversification of traits in East Asians.

**Conclusions:**

Our findings provide a comprehensive genomic dataset that elucidates the fine-scale genetic history and evolutionary trajectory of natural selection signals and disease susceptibility in Han Chinese populations. This study serves as a paradigm for integrating spatiotemporally diverse ancient genomes in the era of population genomic medicine.

**Supplementary Information:**

The online version contains supplementary material available at 10.1186/s12864-024-10514-9.

## Background

East Asia, a center of agricultural domestication during the Neolithic transition, was inhabited by anatomically modern humans at least 50,000 years ago (kya) [1, 2]. This region boasts a complex demographic history and a differentiated genetic architecture of complex traits. A comprehensive understanding of human evolutionary history is essential for elucidating the formation of modern humans and the impact of genetic variation on traits and diseases. These processes include archaic introgression, multiple human dispersal events, admixture, and natural selection [3, 4]. Recent studies utilizing ancient genomic resources have revealed that human expansion from regions such as the Mongolia Plateau, Amur River Basin, Yellow River Basin, Yangtze River Basin, and the Eurasian Steppe has gradually facilitated complex patterns of population structure and genetic diversity in East Asia [5–8]. Evolutionary reconstructions among ethnolinguistically modern populations suggest that differentiated selective pressures and admixture landscapes have enriched the complexity of the population history and biological adaptations of East Asians [6, 9–12]. Yang et al. identified genomic substructures between ancient northern East Asians (ANEAs) and ancient southern East Asians (ASEAs). They also highlighted coastal population migrations and connections from the Russian Far East, coastal China, and Vietnam from the late Pleistocene to the Holocene epoch [13]. Research in population genomics has indicated that complex demographic processes such as effective population size, divergence times, isolation, and migrations are shaped by gene flow between early highly differentiated populations or barriers to forming cultural and ecological differences. Genetic components derived from distinct ancestral sources have increased genomic diversity and phenotype complexity. Post-admixture adaptation in new environments may further influence gene expression profiles and phenotypic traits [14]. Consequently, East‒West admixed populations in Central Asia and northwestern Chinese Turkic-speaking people possess unique adaptive landscapes of critical immune and metabolic pathway genes [15]. Environmental factors, including dietary shifts and regional living conditions, have also shaped adaptive variants. Yang et al. dissected the genetic basis of the skin color phenotype in highland East Asians and reported that the darker baseline skin color in Tibetans was induced by a mutation (rs75356281) under adaptation to strong ultraviolet (UV) radiation [16].

In recent decades, extensive genomic studies have explored genetic diversity within European populations, aiming to uncover how genetic disease risks originate from various geographically isolated conditions. FinnGen, a large-scale biobank resource, utilizes isolated populations to identify disease-predisposing alleles prevalent in Finland [17]. The underrepresentation of non-European populations in global human genomic resources and clinical genomic datasets remains a significant concern. Although genomic projects in East Asia, such as GenomeAsia100K [18], Nyuwa [19], and ChinaMAP [20], have played significant roles in discovering genotype‒phenotype associations, the representation of genetic diversity remains limited. More fine-scale genomic projects are needed to focus on genetic variation discovery, population history reconstruction, and medical relevance interpretation to dissect the genetic landscape of Chinese populations. Current human genetic studies have identified numerous population- or region-specific genetic variants associated with disease susceptibility. The origins of many common and rare genetic diseases are traced to specific ancient lineages and genetic adaptations. In the immune system, the evolution of host‒ interactions enhances the ability to resist infections but also increases susceptibility to inflammatory diseases [21]. This evolutionary pattern, known as antagonistic pleiotropy, provides fitness benefits for survival in extreme environments but also elevates disease burden. Generally, the physical environment, dietary practices, and exposure to endemic pathogens are persistent driving factors of natural selection and adaptation mechanisms. These factors impose heterogeneous selection pressures on humans, leading to differential, adaptive genes and significant variations in the prevalence of genetic diseases among populations with diverse genetic backgrounds.

Han Chinese, the world's largest ethnic group, is traditionally distributed across seven geographical regions and is divided into two genetically distinct groups: northern Han and southern Han [22]. Previous genetic studies utilizing genome-wide SNP variations, mitochondrial DNA (mtDNA), and Y-chromosomal variations have demonstrated a North‒South population structure and varying allele frequencies between northern and southern Han people [23–25]. Furthermore, whole-genome sequencing analyses have identified finer subgroups within Han Chinese individuals, attributable to the population's complex origins and historically documented interactions with surrounding ethnic groups [20, 26]. The ancestral origins of Han Chinese people remain a subject of ongoing debate. Zhang et al. proposed that the homeland of the Proto-Sino-Tibetan (Proto-ST) language originated in the YRB of northern China, supporting the northern-origin hypothesis of the ST people [27]. The YRB civilization, which encompasses cultures such as Yangshao, Majiayao, and Longshan, significantly influenced extensive regions and gave rise to present-day Han Chinese culture [28, 29]. The genetic patterns observed in northern Han Chinese also have been partly attributed to millet-based farming populations from the West Liao River (WLR) region in northeastern China. The expansion of farming practices increased the genetic affinities between WLR millet farmers and YRB ancients during the late Neolithic period. However, the influence of YRB-related ancestry decreased in Bronze Age populations due to changes in subsistence strategies [30]. The agricultural systems in China exhibit a dualistic structure, with rice domestication first documented at the Kuahuqiao and Xianrendong sites in the YZRB [31, 32]. The coexistence of millet and rice farming at various archaeological sites indirectly provides evidence for North‒South gene flows [33, 34]. In addition to the genetic substructure in the North‒South direction, East‒West differentiation is also present among Han Chinese individuals. Genetic evidence indicates that massive demic diffusion, the North‒South admixture model, and trans-Eurasian complex admixture have all shaped the genetic profile of Han Chinese [2, 22, 25, 35].

Shandong is located on the Shandong Peninsula along the eastern coast of China in the lower Yellow River Basin (YRB), a core region of the ancient millet domestication center and Neolithic transition hotspots. This area faces the Bohai Sea and is situated across the sea from the Korean Peninsula and the Japanese archipelago. Numerous Neolithic cultures, such as the Houli [36], Dawenkou [37], and Longshan [38] cultures, have been identified in this region. Yang et al. illustrated that the genetic background of coastal early Neolithic ANEA in Shandong differed from that of inland Neolithic Yumin and Fujian Neolithic ASEA [13]. Archaeological and genetic evidence has revealed that the late Neolithic society in ancient Shandong Province followed a matrilineal community structure, exhibiting low mitochondrial DNA (mtDNA) diversity but high Y chromosome diversity [37]. By analyzing the appearance of haplogroups C, M9, and F, Liu et al. reported that maternal genetic structure began to change 4600 years before the present (BP) and that the ancestral components of the Bianbian individuals were related to ANEA and ancient Siberian lineages [38]. However, comprehensive population genetic studies of modern populations in the Shandong Peninsula, such as the Shandong Han (SDH) population, are lacking. Additionally, research on the genetic connection between geographically close ancient and modern populations is limited due to the sparse sampling of present-day individuals. The genetic origins of the Han Chinese people in the YRB, the phylogenetic relationships between the northern Han people and their geographical neighbors, and their biological adaptation signatures remain poorly characterized. Investigating the fine-scale genetic structure of the northern Han people in the YRB can offer unique insights into specific disease evolutionary events. To address these gaps, SDH was chosen as a representative population of the Northern Han population. An extensive population genetic analysis was conducted, incorporating data from other Northern Han populations located in Shanxi, Henan, and Shaanxi provinces. This study aimed to investigate the northern Han population structure, identify biological adaptive signals, and reveal the evolutionary origins of East Asian-specific diseases using ancient and modern genetic data. A total of 264 Han individuals from various cities in Shandong Province were genotyped using an Affymetrix array (Supplementary Fig. 1a). The genome-wide single nucleotide polymorphism (SNP) data of SDH were integrated with those from spatiotemporally diverse ancient East Asians and other linguistically diverse modern East Asian populations, including the Altaic, Tai-Kadai (TK), Hmong-Mien (HM), Austronesian (AN), Austroasiatic (AA), and Sino-Tibetan (ST) groups. This research illuminated the impact of genetic interactions between ancient and modern East Asians on the gene pool of SDH and explored the admixture processes and adaptation mechanisms of SDH. Changes in allele frequencies associated with traits over time were observed, and the trajectories of allele frequency changes at highly differentiated variants (HDVs) are depicted. By comparing ancient genomes spatiotemporally, valuable insights were provided into the genetic continuity and mobility of Han Chinese from the YRB lineage.

## Results

### Genetic structure and population affinity

**Fig. 1:**
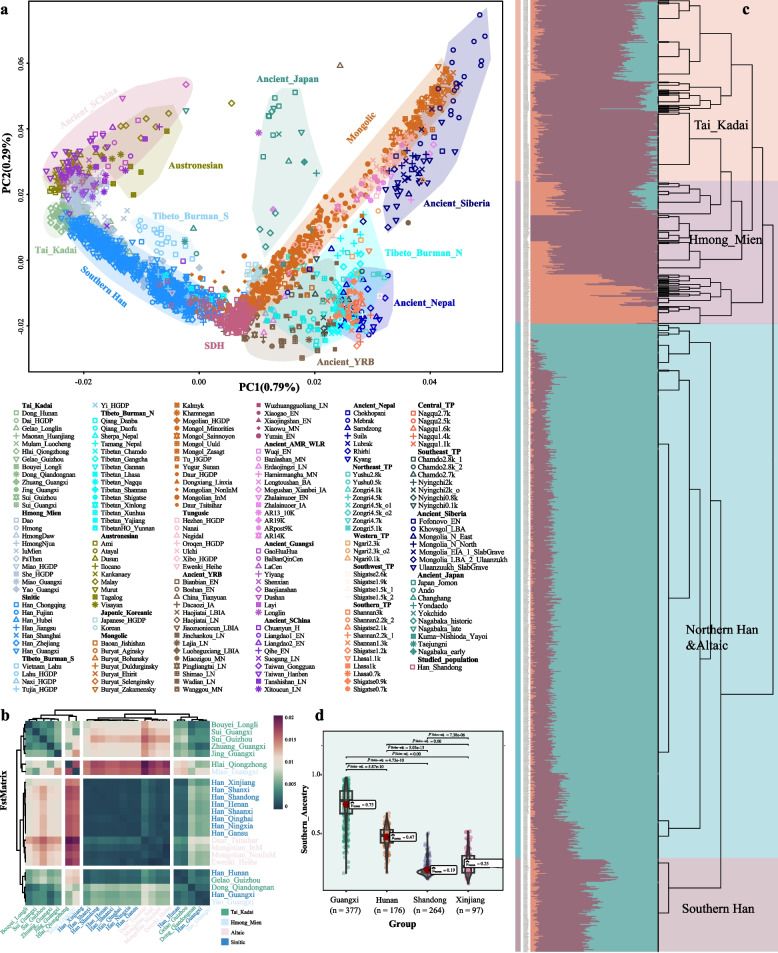
Genetic affinity and differentiation of geographically different modern and ancient East Asian populations and surrounding populations.** a **Principal component analysis of 22 modern and ancient East Asians based on merged low-density genomic datasets. The studied population is colored red, and clusters or clines of other reference populations are labeled on the plot in different colors. **b** The pairwise *F*_ST_ genetic distances between the Shandong Han (SDH) population and 23 ethnolinguistically different groups. **c** FineSTRUCTURE revealed the phylogenetic topology of 24 East Asian populations at a fine scale based on the Affymetrix dataset. The ADMIXTURE results indicated that the best-fit model of the above populations was at K = 3 based on the same dataset. **d**. Interpopulation comparisons of the proportions of southern ancestral components in the four Han populations inferred from ADMIXTURE analysis are shown in Supplementary Fig. 1f

To elucidate the general genetic relationships between SDH and other East Asians, we conducted an integrative analysis using genome-wide data from both modern and ancient individuals. The Human Origin (HO) and Affymetrix datasets were merged, resulting in a new low-density dataset (Affy_HO). Principal component analysis (PCA) was performed in the East Asian context, and ancient individuals were projected onto the genetic backgrounds of modern populations based on the merged Affy_HO dataset. PCA revealed that SDH lies along the axis between the northern and southern Chinese populations and is located in the northern genetic cline, partly overlapping with southern Han and Mongolians (Fig. 1a). Notably, Tungusic-speaking populations simultaneously tended to shift toward SDH, indicating their potential genetic interactions. Moreover, ancient individuals from the YRB, including Dacaozi, Jiaozuoniecun, and Pingliangtai, displayed apparent genetic affinities with SDH. Some SDH individuals deviated toward southern Han Chinese and were clustered closer to modern Japanese and Korean populations than were ancient people, reflecting additional gene flow events.

To better understand the genetic structure of SDH, we further investigated the genetic affinity and differentiation among geographically distinct Han Chinese and minority ethnic groups. Our findings revealed genetic differences among geographically different Han populations along latitudinal and longitudinal gradients. Based on the pairwise fixation index (*F*_ST_) estimation, we observed that SDH had the closest genetic relationships with the northern Han population, followed by the surrounding minority ethnic groups (Fig. 1b). However, interestingly, SDH exhibited a more distant genetic relationship with the Guangxi Han (GXH) population than with the northern Altaic-speaking populations, such as the Mongolian, Ewenki, and Daur populations. Additionally, we found that the genetic differences between the SDH and Xinjiang Han populations (XJH, *F*_ST_ = 0.0001) were not as apparent as those between the SDH and GXH populations (*F*_ST_ = 0.005). GXH represents the southern Han population, and XJH lives in the westernmost region of China. The *F*_ST_ results indicated that genetic differentiation among Han populations was more prominent in the North‒South region than in the East‒West region (Supplementary Table 1). Hierarchical clustering patterns inferred from TreeMix and identity by descent (IBD)-based heatmap analyses revealed that Han people could be divided into subgroups based on their geographical origin and affinity. There was one case of gene flow from XJH to GXH in the phylogenetic tree. However, no apparent gene flow events related to SDH were detected (Supplementary Fig. 1c). Furthermore, the estimated pairwise IBD fragments showed that SDH had the closest genetic affinity to Shanxi Han, and the shortest shared IBD segments were observed between SDH and southern TK-speaking Hlai people from Qiongzhong in Hainan Province. Compared with other populations, the genetic affinity of the Han populations from northern and northwestern China was greater (Supplementary Fig. 1d). The runs of homozygosity (ROH) of 24 geographically different populations also showed genetic differentiation among them (Supplementary Fig. 1e). Moreover, we also conducted haplotype-based fineSTRUCTURE analyses to explore the fine-scale genetic structure among 1061 individuals from various regions across China. We observed four major genetic clusters in the haplotype-based dendrogram and identified a significant North‒South gradient of genetic components related to different ancestral sources (K = 3, Fig. 1c). To illustrate the significant differences in the genetic components of geographically distinct Han Chinese populations, we conducted interpopulation comparisons focusing on southern ancestral components. Specifically, we selected Hunan Han (HNH) and GXH as representative southern Han populations. Furthermore, we included XJH in the population admixture model to represent the Han population in the westernmost region of China (Supplementary Fig. 1f). Statistical indices further demonstrated significant differences in southern ancestral components among the four Han populations from different geographical locations. These findings supported the coexistence of a North‒South structure alongside an East‒West cline, which aligned with the admixture model mentioned above (Fig. 1d). The observed patterns of genetic clustering indicated that Han Chinese could be separated into geography-related population stratifications.

### Ancestral makeup and admixture landscape of Han Chinese from the lower YRB

**Fig. 2:**
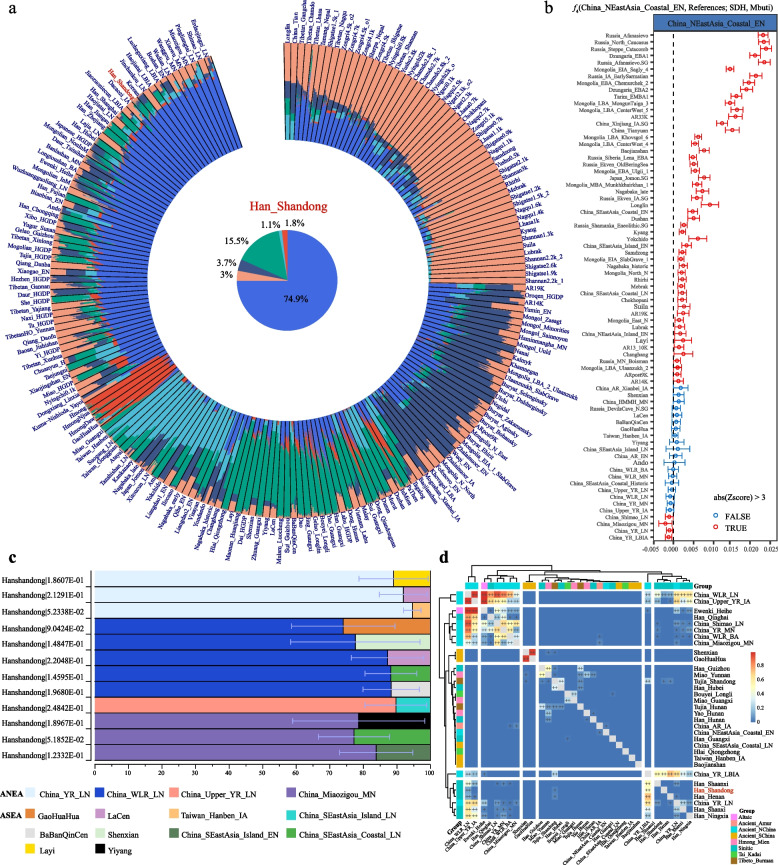
Admixed landscape and demographic history of the SDH population.** a** ADMIXTURE results for the SDH population and 201 reference populations at K = 6. Different colors represent different ancestral sources in the clustering patterns. **b** The *f*_4_-statistics in the form of *f*_4_(Ancient Reference1, Ancient Reference2; SDH, Mbuti) were calculated to test the genetic affinity between SDH and ancient references. Ancient Reference1 and Ancient Reference2 represented 76 selected ancients in East Asia. |Z|> 3 is indicated by TRUE, indicating closer genetic affinity between SDH and Ancient Reference2. **c** The qpAdm-estimated admixture proportions of different ANEA and ASEA in SDH. We used two-way admixture models to determine the genetic contributions of ANEA and ASEA. The ANEA included YRB-related farmers and ancient individuals from the WLR. The ASEA mainly consisted of ancient people from Guangxi and Fujian. **d** Pairwise qpWave results show the genetic heterozygosity between SDH and ancient/modern humans in East Asia based on the merged Affy_1240K dataset

We used an unsupervised model-based ADMIXTURE analysis of SDH combined with modern and ancient East Asians to dissect the ancestry makeup of SDH and explore the genetic influence of the surrounding populations (Fig. 2a). The best-fit admixture model with six ancestral sources revealed that compared with other Han people, the SDH population harbored a greater proportion of YRB-related ancestral components (dark blue). Ancient Siberian-related components decreased from ancient Shandong people (e.g., Bianbian, Xiaogao, and Boshan) in the Neolithic Age to modern SDH, while ancient YRB-related ancestry components increased. Furthermore, our findings indicated that southern East Asian ancestry was greatest in TK-speaking populations (dark green), HM-speaking populations (red), ancient Tibeto-Burman (TB) populations from the southern Tibetan Plateau, and ancient Hanben populations. Siberian-dominant ancestry was essential for the formation of the gene pools of Altaic people, Japanese people, Koreans, and northern Chinese Sinitic speakers, which gradually became rare or absent in southern East Asian populations, such as HM and TK speakers.

We used various ancestral source pairs to investigate the genetic contribution to SDH via admixture-*f*_3_(Source1, Source2; SDH), in which significant negative Z scores (Z < -3) indicated prominent admixture events. As shown in Supplementary Fig. 2a, we observed that the combination of ANEA associated with late Neolithic Longshan millet farmers (China_Upper_YR_LN) and southern ancestry linked to the Iron Age Hanben people (Taiwan_Hanben_IA) resulted in a negative *f*_3_-value (Z = -3.65, Supplementary Table 2). The ANEA and ASEA pairs could serve as possible ancestral sources of SDH, consistent with the mosaic ancestry composition observed in the ADMIXTURE-based results. We validated the North‒South admixture model using modern populations (Supplementary Fig. 2b and Supplementary Table 3). In this model, the northern source comprised Altaic-speaking populations (Mongolian and Daur) and Sinitic speakers, while the other source encompassed southern East Asians associated with HM/TK-speaking populations, as estimated by *f*_3_(Altaic, southern East Asians; SDH). Additionally, we investigated the genomic affinity between the target population and other ancient Asians via affinity  *f*_4_-statistic of *f*_4_(Ancient Reference1, Ancient Reference2; SDH, Mbuti). Our results revealed that SDH shared more alleles with ANEA than with ASEA. Compared with ancient individuals in Shandong (China_NEastAsia_Coastal_EN), a stronger genetic affinity was observed between modern SDH and four ancient populations in the upper and middle YRB (China_YR_LBIA, China_YR_LN, Shimao, and Miaozigou), suggesting the apparent genetic influence of ancient Yangshao and Longshan people on modern Shandong people (Fig. 2b; Supplementary Table 4). Therefore, based on the affinity *f*_4_-statistics, it can be inferred that the gene pool of SDH has been more affected by YRB-related farmers and ancient individuals in Inner Mongolia since the early Neolithic period. To further investigate whether SDH descended directly from ANEA, we computed *f*_4_(ANEA, SDH; Reference, Mbuti). Only a few significant signals (indicated by a red color with a Z score > 3) were observed when we assumed that Malaysia_LN and Laos_LN_BA were the reference ancestral sources (Supplementary Fig. 3a and Supplementary Table 5). Thus, compared to China_NEastAsia_Coastal_EN, SDH obtained additional gene flows from late Neolithic Malaysians and Laotians during the late Neolithic to Bronze Age. However, we should also pay attention to these two weak signals, which might be caused by the low overlap of SNPs and ancient DNA damage signals. Furthermore, significant negative *f*_4_ values were observed for Taiwan_Hanben_IA and GaoHuaHua when we assumed that China_YR_MN was the ancestral contributor in the form of *f*_4_(China_YR_MN, SDH; Reference, Mbuti) (Supplementary Fig. 3b). As shown above, most of the results exhibited non-significant *f*_4_-values (|Z|≤ 3), and several significant *f*_4_ values indicated that southern ancestral populations also contributed genetic materials to SDH.

We then used asymmetric *f*_4_(ASEA, SDH; Reference, Mbuti) to explore additional possible ancestral sources for the formation of SDH. When we hypothesized that Hanben was their possible ancestor, SDH obtained more gene flow from ancient reference populations in the Mongolian Plateau, Siberia, Nepal, and WLR/ARB/YRB than did ASEA, suggesting that ANEA contributed significantly to the modern SDH's gene pool (Supplementary Fig. 3c and Supplementary Table 6). The statistically significant negative values observed in *f*_4_(ANEA, SDH; ASEA, Mbuti) were also consistent with the *f*_*3*_-based admixture models, in which ASEA shared more alleles with SDH than non-YRB ANEA surrogates. Moreover, the extent of genetic heterogeneity between the SDH population and the southern Han populations is still worth investigating. We used *f*_4_-statistics in the form of *f*_4_(Southern Han, SDH; Reference, Mbuti), aiming to test their genetic differences and which factors contributed to differentiation (Supplementary Table 7). SDH did not form one clade with southern Han Chinese individuals, as indicated by the statistically significant negative and positive *f*_4_ values. These statistically significant values further indicated that the northern Han and southern Han peoples experienced different genetic influences from their geographically close indigenous neighbors in terms of their past demographic processes, including early divergence and subsequent extensive admixture or gene flow events with other sources (Supplementary Fig. 4a-c). More alleles related to Mongolic, Tungusic, TB speakers, and Western Eurasians were detected in the SDH than in the southern Han populations from Hunan and Chongqing Provinces. We further tested the admixture models and quantified the proportions of admixtures with different ancestral sources at different scales using qpWave and qpAdm analyses. The well-fit two-way admixture model suggested that SDH can be simulated as a North‒South admixture model. In this model, YRB-related ancestries, including Miaozigou_MN, Upper_YR_LN, and YR_LN, represented the ANEA source. The ASEA component comprised SEastAsia_Coastal_LN, BaBanQinCen, and Taiwan_Hanben_IA. The average estimated genetic contributions of ANEA and ASEA were approximately 0.85 and 0.15, respectively (standard error of the mean = 0.11, Fig. 2c and Supplementary Table 8). We observed that the greatest proportion (0.946 ± 0.026) of ANEA contributed to the gene pool of SDH when the geographically close Longshan people served as northern ancestral sources and IronAge Hanben served as southern ancestral sources, consistent with the clustering patterns of the relative genetic stability observed in ADMIXTURE and PCA. Based on the admixture-induced linkage disequilibrium for evolutionary relationships (ALDER), we further estimated the admixture time of SDH based on the LD decay pattern. The results revealed that the northern Han exhibited genetic contact with Altaic-speaking Yakut approximately 101 generations ago, aligning with the late Shang Dynasty and the Western Zhou Dynasty (Supplementary Table 9). The estimated ancient genetic connection with Siberians supported persistent cultural and population communication or contact between YRB farmers and ancient Siberians. Early Neolithic Yumin people from the Mongolian Plateau; Neolithic Boshan, Xiaogao, Xiaojingshan, and Bianbian people from Shandong Province all possessed a close genetic connection with ancient Neolithic Siberian lineages [13].

### Relative genetic stability and temporal dynamics in the lower YRB since the Neolithic period

According to the *f*_4_-statistics mentioned above, SDH received a relatively low degree of genetic influence from non-YRB lineages and maintained a high level of genetic stability. These findings indicated genetic continuity within this region since the Neolithic period. Inferring spatiotemporal patterns of genetic change in populations from the YRB would favor dissecting the formation of northern Han Chinese. We first conducted PCA in the context of ancient East Asians, and modern people were projected onto the first two PCs. We found that northern Han populations in the YRB formed a tight cluster and exhibited genetic similarity with geographically close ANEAs (Supplementary Fig. 5a). The PCA results demonstrated that present-day Han people in the lower YRB overlapped with ancient YRB millet farmers and reflected a considerable degree of genetic affinity between them. We utilized a series of *f*_4_-statistics to investigate temporal patterns of genetic continuity among the YRB-related populations, spanning from the early Neolithic era to modern times (Supplementary Table 10). Our findings revealed that early Neolithic coastal inhabitants in the lower YRB (China_NEastAsia_Coastal_EN) were more substantially affected by ancient people from Siberia and the Mongolian Plateau than were the Middle Neolithic Yangshao people (YR_MN, Supplementary Fig. 6a). Gene flow between coastal and inland Neolithic ANEAs was inferred from positive values in *f*_4_(China_NEastAsia_Coastal_EN, China_YR_MN; Reference, Mbuti). During the late Neolithic period, we found that the component related to the ASEA increased based on *f*_4_(China_YR_MN, China_YR_LN; Reference, Mbuti). Late Neolithic Longshan individuals (China_YR_LN) gradually shared more alleles with the ASEA than with the YR_MN, consistent with the northward expansion of rice farmers (Supplementary Fig. 6b). However, there were no further significant genetic shifts from YR_LN to the late Bronze/Iron Age (YR_LBIA), as evidenced by *f*_4_(China_YR_LN, China_YR_LBIA; Reference, Mbuti) (Supplementary Fig. 6c). We then carried out *f*_4_(YR_LBIA, SDH; Reference, Mbuti) to assess the genetic homogeneity between the late Bronze/Iron Age individuals and present-day SDH individuals (Supplementary Fig. 7a). Except for the early and middle Neolithic YRB-related populations, the *f*_4_ values were not significant for any of the tested individuals (|Z|≤ 3). Many nonsignificant *f*_4_ values supported that SDH harbored a strong genomic affinity for YRB-related late Bronze/Iron Age individuals and was less influenced by additional genetic material. Our results revealed clear indications of long-term genetic continuity in the lower reaches of the YRB since the early Neolithic, with modern SDH still displaying high genetic homogeneity with YRB-related ancestors.

Similarly, the pairwise qpWave analysis focused on YR_LBIA and SDH was consistent with the findings of the *f*_4_-statistics and further confirmed their genetic homogeneity (Fig. 2d). Moreover, the values of qpWave validated that YR_LBIA was also genetically close to other Han populations in the lower reaches of the YRB (e.g., Shanxi, Henan, and Shaanxi). However, genetic heterogeneity existed among the four Han populations in the YRB. The *f*_4_(YR_LBIA, Han Shanxi/Henan/Shaanxi; Reference, Mbuti) explained in detail which gene flows caused the differences (Supplementary Fig. 7b-d). On the one hand, Han Chinese individuals from Shaanxi, Henan, and Shanxi Provinces shared more alleles with non-YRB ancestral sources than did those from SDH, represented by DevilsGate hunter-gatherers, Mongolic speakers, and WLR-related populations. On the other hand, gene flows from the ASEA and ancient East/Southeast Asians, such as China_SEastAsia_Coastal_LN, Gaohuahua, and Vietnam_BA, all contributed to the increasing genetic diversity of Han Chinese from Shanxi and Shaanxi provinces. We then evaluated the demographic processes of the four Han populations by effective population size (Ne, Supplementary Fig. 5b). SDH steadily increased before 25 generations, and Shanxi Han experienced a bottleneck effect at approximately 10 generations. However, the height of the Han populations from Henan and Shaanxi increased faster than that of the former. Our analysis suggested that migration from southern populations has exerted less influence on SDH since the Bronze Age, which may serve as one possible interpretation of the genetic stability observed in SDH.

### Natural selection and adaptation mechanisms

**Fig. 3:**
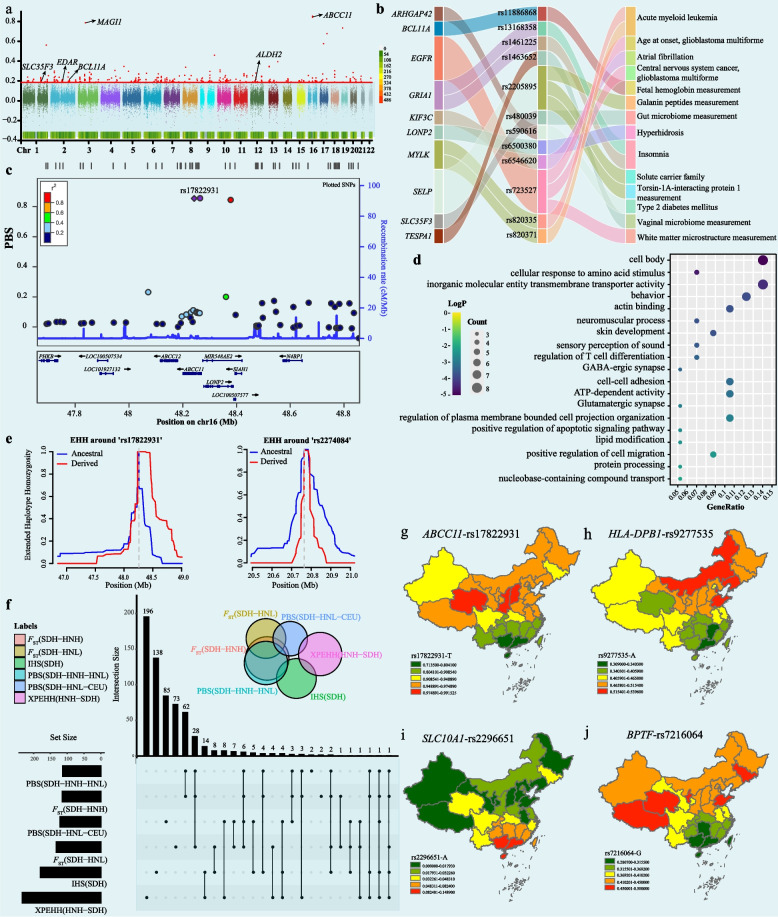
Biological adaptation signals in northern Han people.** a** The Manhattan plot shows the natural selection signatures for SDH. The top 0.1% of the PBS values above the red line were identified. **b **The Sankey diagram reflects gene pleiotropism. **c** LocusZoom provided the regional visualization of *ABCC11* variants in the top 0.1% of PBS values. **d** KEGG and GO enrichment analysis of the top 0.1% of the candidate genes identified by PBS (SDH-HNL-CEU trio model). **e** Extended haplotype homozygosity of two SNPs (rs17822931 and rs22744084) selected by PBS. **f** Venn diagrams showing the shared selection-nominated candidate genes of the SDH based on different methods. **g-j** Adaptative allele frequency and derived allele frequency distribution of highly differentiated variants among Chinese populations

Characterizing the biological adaptability of genetically distinct populations is essential for understanding the evolutionary driving forces of complex genetic traits or diseases. We first used population branch statistics (PBS) to explore significant natural selection signatures in the SDH to dissect population-specific variants. We used SDH as the target population and selected Hlai_Qiongzhong (HNL) and Europeans (CEU) as the ingroup and outgroup reference populations, respectively. In the SDH-HNL-CEU trio model, we detected 340 SNPs in the top 0.1% (PBS > 0.185) of the mean genome-wide PBS scores (Fig. 3a and Supplementary Table 11). As a supplement to PBS, we also applied the integrated haplotype score (iHS) and *F*_ST_ to further validate these biological adaptive signals in the SDH (Supplementary Tables 12–13). Combined with haplotype and allele frequency methods, we identified 159 high-quality variants, 19.5% of which were missense variants (Fig. 3f). The SDH-specific selection signals identified by iHS were primarily associated with height (*PDHX*, pyruvate dehydrogenase component X), atopic asthma (*TAP2*, Transporter 2), and BMI-adjusted waist-to-hip ratio (*HLA-B*, major histocompatibility complex; *HLA-C*, *L3MBTL3*, L3MBTL histone methyl-lysine binding protein 3 and *C6orf10*, testis expressed basic protein 1).

Among the 340 candidate SNPs, the strongest PBS value was observed for ATP binding cassette subfamily C member 11 (*ABCC11*), which is located on chromosome 16 (Fig. 3c). The *ABCC11* variant is associated with human axillary odor (AO) and earwax type [39]. The ancestral allele (rs17822931-G) was demonstrated to increase the risk of axillary osmidrosis, while the derived allele (rs17822931-A) displayed a positive selection signal in East Asians, suggesting that it may confer an adaptive advantage in cold climates [40]. This polymorphism has undergone a complex evolutionary history, resulting in diverse allele frequencies across spatiotemporally different ancient and modern populations. Previously identified natural selection signals, including Ectodysplasin A receptor (*EDAR*) [41], Solute carrier family 35 member F3 (*SLC35F3*) [42], and acetaldehyde dehydrogenase 2 (*ALDH2*) [43], have been implicated in phenotypic traits such as shovel-shaped incisors, thiamine metabolism, and alcohol metabolism. We subsequently annotated the 159 robust SNPs via the Variant Effect Predictor (VEP) and Genome-Wide Association Study (GWAS) catalogs. A Sankey diagram indicated that candidate genes for selection and their associated phenotypes exhibited potential pleiotropy (Fig. 3b). For instance, rs723527, which is associated with epidermal growth factor receptor (*EGFR*), influences central nervous system cancer and glioblastoma multiforme and participates in white matter microstructure measurements. *ARHGAP42* (rs590616, Rho GTPase Activating Protein 42) has been identified as a risk factor for type 2 diabetes mellitus. Additionally, two variants within the glutamate ionotropic receptor AMPA type subunit 1 (*GRIA1*) gene, rs13168358 and rs1461225, were associated with insomnia. *GRIA1* encodes an excitatory receptor for neurotransmitters and has been linked to deficits in short-term habituation, as demonstrated in animal models [44]. This gene was also subject to strong selection in the studied population, second only to *ABCC11*, as evidenced by iHS and PBS scores. The *BCL11A* (*BCL11* transcription factor A) variant rs11886868 is crucial for regulating fetal hemoglobin (HbF) levels and inhibiting deoxy sickle hemoglobin polymerization. Downregulating *BCLL11A* expression is a promising therapeutic strategy for sickle cell disease [45, 46]. The ancestral allele of this variant is T, and the derived allele is C, of which the CC genotype can promote HbF induction and ameliorate the repression effect on γ-globin in patients with sickle cell anemia [47]. GO and KEGG enrichment analyses revealed that the candidate genes were primarily enriched in pathways pertaining to the cell body and cellular response to amino acid stimulus (Fig. 3d).

Different methods can detect different timescale biological adaptation signatures [48]. Pairwise fixation index (*F*_ST_) estimation between SDH and HNH was used to examine recent natural selection signals (Supplementary Table 14). The top 0.1% of *F*_ST_ results were detected as candidate loci, but this method cannot determine whether natural selection signals occurred in SDH or HNH. We conducted PBS again and chose HNH and HNL as the second and third reference populations, respectively (Supplementary Fig. 8a and Supplementary Table 15). Overall, 193 SNPs of the top 0.1% of selection signals identified in the PBS group were also supported by the cross-population extended haplotype homozygosity (XP-EHH) approach and *F*_ST_ (Fig. 3e-fand Supplementary Table 16). The gene encoding *CACNA1A* (rs16029, calcium voltage-gated channel subunit alpha1A), a gene implicated in regulating calcium ion entry into excitable cells and neurotransmitter release within the nervous system, was expressed at the highest level in the PBS-treated group. Additionally, six other genes demonstrating adaptive signatures were identified: *LILRA3* (leukocyte immunoglobulin-like receptor A3), *MTHFR* (methylenetetrahydrofolate reductase), *GJB2* (gap junction protein beta 2), *FADS1* (fatty acid desaturase), *FADS2* and *KRT14* (keratin 14). Notably, *LILRA3*, a leukocyte immunoglobulin-like receptor (LILR) family member, plays a role in the immune response [49]. The selected mutation locus (rs410852) within the *LILRA3* gene can increase Takayasu arteritis (TAK) susceptibility, which is inferred from GWAS catalog data. TAK is predominantly prevalent in East Asians and is potentially linked to ethnic background [50]. The identified mutation can stimulate proinflammatory cytokine production and induce the proliferation of specific immune cell types [51]. *The MTHFR* gene encodes a methylenetetrahydrofolate reductase and maintains the balance between methionine and homocysteine [52]. Two polymorphisms within *MTHFR*, rs1801133 and rs9651118, are under natural selection pressure. Notably, rs1801133 is the most common genetic determinant for methylenetetrahydrofolate reductase deficiency, a condition that heightens the risk of cardiovascular disease [52]. Moreover, rs9651118 is linked to moyamoya disease and red blood cell distribution width changes. Two *GJB2* gene mutations, rs72474224 and rs2274084, were associated with nonsyndromic hearing loss. The rs72474224 variant (c.109G > A, p.V37I) is especially prevalent in East Asians and confers a substantial genetic predisposition to hearing impairment [53]. Additionally, our analyses further revealed other signals of selective sweeps in genes associated with diverse phenotypic traits (Supplementary Fig. 8b), including skin pigmentation (*MC1R*, melanocortin 1 receptor), stature (*VPS9D1*, *VPS9* domain containing 1), and smoking behavior (*SLC38A3*, solute carrier family 38 member 3). Enrichment analyses suggested that these adaptive genes were involved in biological processes such as transport across the plasma membrane, phosphotransferase activity with alcohol groups as acceptors, and immune receptor activity (Supplementary Fig. 8c).

### Highly differentiated variants between northern Han and southern Han people

Population structure and selection pressure can produce HDVs. F_*ST*(SDH-HNH)_ results were used to identify variants with significant differences in allele frequency between the northern and southern Han populations. Based on the top 0.1% of *F*_ST_ values, our findings initially revealed that these HDVs are mainly associated with endemic pathogen exposure and dietary habits, exemplified by genes such as *LILRA3*, *CR1* (complement receptor 1) and *FADS*. *CR1* is associated with malaria resistance, whereas the FADS gene family participates in fatty acid metabolism [54]. Their frequency distribution varied among different populations, with high frequencies observed in southern populations (Supplementary Fig. 8d-g). A variant of particular interest, rs2296651 (c.800C > T, p.Ser267Phe), which is located in solute carrier family 10 member 1 (*SLC10A1*), is implicated in abnormal bilirubin metabolism. *SLC10A1* encodes Na-taurocholate cotransporting polypeptide (NTCP), which facilitates the transport of conjugated bile acids into hepatocytes [55]. The S267F variant leads to NTCP deficiency, and its clinical manifestations include indirect hyperbilirubinemia and transient cholestatic jaundice [56]. Moreover, *SLC10A1* serves as a receptor for the hepatitis B virus (HBV), and this variant can increase resistance to chronic hepatitis B [57, 58]. Information from the GWAS catalog revealed that the rs2296651 mutation was associated with alterations in the levels of multiple metabolites, including glycocholic acid, low-density lipoprotein cholesterol, and total cholesterol. The highest allele frequency for this variant was observed in East Asians according to the gnomAD database, with the loci under natural selection confirmed by the XP-EHH method (Supplementary Fig. 9f). Our study also revealed other complex relationships between HDVs and disease susceptibility. For instance, we identified two variants in the *BPTF gene*. The rs12602912 variant is associated with body mass index, and rs7216064 determines genetic susceptibility to lung adenocarcinoma and lung carcinoma.

### Haplotype analysis and allele frequency trajectories of *ABCC11 *and* SLC10A1*

**Fig. 4:**
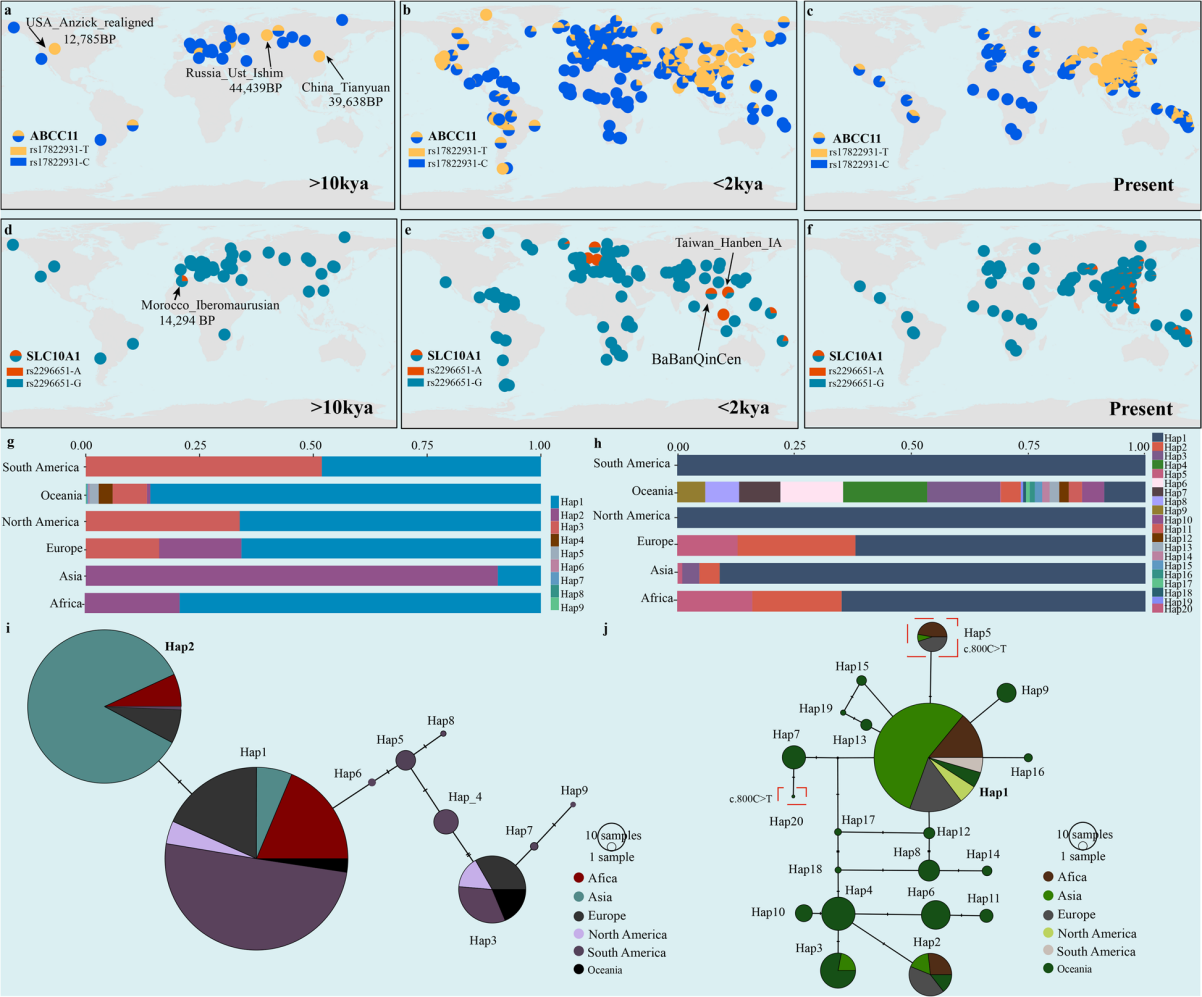
Evolutionary trajectories and haplotype analyses of *ABCC11* and* SLC10A1*.** a-c** Allele frequency distribution of rs17822931-T across global populations in various spatiotemporal contexts, encompassing time points approximately 10,000 years ago, nearly 2000 years ago, and the present day. The detailed evolutionary trajectories from 10,000 years to 2000 years are presented in Supplementary Fig. 8 h-j. d-f Allele frequency distribution of rs2296651-A across global populations in various spatiotemporal contexts, including time points approximately 10,000 years ago, nearly 2000 years ago, and the present day. The detailed evolutionary trajectories are presented in Supplementary Fig. 8 k-m. **g** Stacked plot showing the haplotype frequencies of the *ABCC11* gene based on the genomic data of the global population. **h** The stacked plot shows the haplotype frequencies of the *SLC10A1* gene based on the same dataset. **i** Haplotype network analysis of 6 SNPs located in the *ABCC11* gene. **j** Haplotype network analysis of 11 SNPs located in the *SLC10A1* gene

The evolutionary histories of numerous genetic variants remain elusive, presenting a barrier to understanding the genetic basis of complex traits or diseases. The *ABCC11* gene serves as a significant biological adaptive signal, determining earwax type and axillary odor. Some hypotheses have posited that the A allele of *ABCC11* (rs17822931) may confer adaptive advantages in colder climates. The ancestral environment of East Asians is thought to have been much colder than that of Africans. The *ABCC11* mutation, characterized by diminished sweat gland activity, has endowed humans with the ability to better preserve body heat in colder climates [40]. Ancient genomic data revealed that the mutation emerged approximately 44,000 years ago in the Ust_Ishim individual rather than in ancient African populations. However, it was less commonly found in populations residing in equatorial regions with higher temperatures two thousand years ago, suggesting a correlation with latitude (Fig. 4a-cand Supplementary Fig. 8 h-j). Subsequently, the mutation also occurred in southern Asia and Oceania for nearly a thousand years, likely attributable to the southward migration of populations from colder northern areas and admixture with indigenous inhabitants. In China, we found that the derived allele of the *ABCC11* gene was first identified in ancient Tianyuan individuals. In North America, rs17822931-T appeared approximately 12,000 years ago. Contemporary East Asians exhibit the highest frequency of this mutation, followed by North American and South American populations. These observations confirmed the association of the mutation with cold environmental adaptation and its status as a region-specific adaptive signal, particularly among East Asians. Indeed, we observed that the frequency of derived alleles was greater in northern Chinese populations due to colder environments (Fig. 3g). We further analyzed the haplotype diversity across global populations and constructed a linkage disequilibrium plot (Supplementary Fig. 9a). We found that Hap2, which carries the derived allele sequence (CTTGCT), was predominantly distributed in Asian people, followed by Europeans (Fig. 4g and 4i). However, Oceanians possessed a distinct haplotype (Hap8, TCTGCT) that contained the derived alleles. Haplotype diversity was constrained among Han populations, with most individuals carrying the derived allele (Supplementary Fig. 9c). The high-frequency haplotype was widespread among minority ethnic groups. However, the AN-speaking populations had different dominant haplotypes, with a comparatively low CTTGCT haplotype frequency.

Although NTCP deficiency is a newly described disease, ancient DNA can provide crucial information about historical allele frequency fluctuations and the geographical distribution of associated mutations. We estimated the temporal origin of the *SLC10A1* mutation (rs2296651) and its historical prevalence by examining allele frequency trajectories over time (Fig. 4d-f and Supplementary Fig. 8k-m). The derived *SLC10A1* variant (rs2296651-A) initially emerged in the Iberomaurusian population of Morocco, with the earliest evidence dating back to approximately 12,849–12,097 calibrated years BCE. This variant was subsequently detected in European populations, such as Italians and Spaniards, and we also found it in the Eneolithic Russia Shamanka circa 7000 to 8000 years ago. The mutation gradually spread eastward, and its presence was discerned in the early period in individuals from California's Channel Islands, where it emerged approximately 4915 years ago. The S267F mutation also appeared in populations from Lebanon and Indonesia during the transition from the Early Bronze Age to the Iron Age. Remarkably, genomic data from Taiwan Hanben revealed the presence of the mutation in ancient southern Chinese individuals approximately 1600 years ago. The derived allele was later identified in ancient Guangxi people (BaBanQinCen) approximately 1400 years ago. Afterward, it was found in Malaysia during its historical period, in which it was composed of Micronesians approximately 580 years ago and Vanuatu people approximately 150 BP. Modern genomic data indicate that the S267F variant is prevalent in Southeast Asian, Oceanian, and southern Chinese populations. Analysis of the allele frequency trajectory across major intercontinental populations suggested a notable increase in the variant approximately 2000 years ago, with a pronounced increase in prevalence in the Asian and Oceanian groups, particularly among coastal southern Chinese populations. NTCP, encoded by the *SLC10A1* gene, is a cellular receptor for HBV and is significantly associated with resistance to chronic hepatitis B [59]. Given the high prevalence of HBV within Chinese populations and the emergence of agriculture in East Asia, we further analyzed the driving force of this biological adaptation [42, 60]. The timeline of mutation emergence allowed us to rule out agricultural development as a driving factor of biological adaptation, instead suggesting a potential link between NTCP deficiency and enhanced pathogen resistance.

Genetic diversity within pathogenic *SLC10A1* allele carrier haplotypes varies in multiple populations. To explore the genetic landscape of the S267F allele carrier haplotypes, we constructed a linkage disequilibrium plot and applied the haplotype-based inference method (Supplementary Fig. 9b). Our analysis revealed 33 haplotypes of the *SLC10A1* gene across six intercontinental populations, with a focus on the primary 20 haplotypes for in-depth analysis (Fig. 4h and 4j). Hap18 (CCGTGAGAAGC) was identified as the ancestral haplotype of *SLC10A1* and is present mainly in Oceanians. However, due to the sparse sampling in Africa, whether ancestral haplotypes exist in Africans remains uncertain. Hap5, characterized by a derived mutation, is widely distributed across Asians, Europeans, and Africans. Conversely, Hap20, which also carried pathogenic genetic variation, was exclusively observed in populations from Oceania. For the S267F mutation, the haplotype (ACGTAAAGGGC) derived from H1 (ACGTGAAGGGC) existed in three linguistically distinct populations, namely, the TK, HM, and TB populations (Supplementary Fig. 9d). Han people predominantly characterized H1, and more diverse haplotypes can be found in other minority ethnic groups, such as the Altaic and AN people.

### Medical relevance

We aggregated all high-quality adaptive loci and HDVs based on the above stringent criteria and evaluated their potential impact on phenotype using three computational prediction methods: Sorting Intolerant from Tolerant (SIFT) [61], Polymorphism Phenotyping (PolyPhen) [62], and Combined Annotation Dependent Depletion (CADD) [63]. Two variants within the *GJB2* gene, rs2274084 and rs72474224, were predicted to be likely damaging, with PolyPhen assigning damage probabilities of 0.998 and 0.995, respectively. Furthermore, the CADD score indicated a relatively high pathogenic risk (exceeding a score of 20) for the rs2274084 (c.79G > A) variant in *GJB2*. The clinical significance of this mutation was categorized as pathogenic or likely pathogenic in the ClinVar database. *GJB2* encodes Connexin26, which is the most crucial gap junction protein in the cochlea. The gap junction system plays an important role in maintaining normal potassium circulation and the microenvironment in the cochlea. These findings all implied that these genetic variations may adversely affect congenital deafness. Intriguingly, our analysis of the minor allele frequency of these two *GJB2* mutations revealed distinct latitude-related distribution patterns. Specifically, the frequency of rs72474224-T was markedly greater in southern populations, while rs2274084-T was more prevalent in northern populations (Supplementary Fig. 9g-h). Additionally, rs11886868 in *BCL11A* was associated with fetal hemoglobin (HbF) levels, with clinical significance deemed benign or likely benign. These findings illuminated the intricate interplay between genetic variations and geographic environment factors, highlighting the multifaceted nature of population genetics.

The *SLC10A1* variant (rs2296651) was predicted to be likely damaged by PolyPhen, with a CADD score also exceeding 20. Furthermore, in line with the American College of Medical Genetics and Genomics (ACMG) guidelines, rs2296651 has been classified as pathogenic or likely pathogenic. The derived allele displayed a greater frequency in southern Chinese populations, especially in people from Guangdong, Guangxi, and Hainan provinces (Fig. 3i). Although the S267F mutation may induce hepatocyte injury due to excessive bile acids, it concurrently provides a protective effect against HBV infection and HBV-related diseases, such as cirrhosis and hepatocellular carcinoma [59, 64]. The A allele is associated with a loss of function in NTCP, thereby interfering with NTCP and HBV binding. More variants with adverse effects have been identified, including rs1801133 in the *MTHFR* gene, which SIFT classifies as deleterious, and PolyPhen as probably damaging, supported by a CADD score above 20. The rs1801133-T locus is associated with decreased *MTHFR* enzyme activity, leading to elevated levels of homocysteine. Notably, hyperhomocysteinemia is a known risk factor for stroke [65]. Similarly, the *CR1* variant rs2274567 is considered to be damaging, according to PolyPhen. *CR1* is an immune receptor that regulates the complement system and is involved in regulating immune responses and clearing immune complexes. This variant may affect the expression level or function of *CR1*, leading to an imbalance in complement system regulation and increasing the risk of some diseases [66]. We further characterized the other two known loci associated with disease traits, delineating their allele frequency disparities (Fig. 3h and j). These candidate loci exhibited differential biological adaptation within Chinese populations, reflecting complex natural selection pressure that can influence genetic predispositions and disease risk discrimination among populations in different regions. Enhancing preventive measures and tailoring clinical interventions for populations with a genetic predisposition to specific diseases is imperative.

## Discussion

Ancient individuals from the YRB and YZRB participated in shaping the genetic landscape of present-day geographically diverse Han populations. The emergence and progression of ancient agriculture along these river systems facilitated population expansion, migration, and admixture [67]. Previous studies have demonstrated that the southward migration of millet farmers influences the genetic profile of southern populations [13]. Furthermore, the northward expansion of rice farmers from the Middle to Late Neolithic period also imparted genetic legacies to the gene pool of northern populations. As one of the agricultural domestication centers of millet farming in China, investigating the genetic structure and population relationships among Han populations residing in the lower YRB at a fine-scale level holds considerable scientific merit. This study explored the intricate genetic interplay between ancient and modern populations and shed light on how these interactions further influenced the genetic makeup of the modern Han. Evidence for a complex demographic history revealed the long-term genetic stability of SDH in the lower YRB. Additionally, numerous East Asian-specific adaptative genetic variants were identified. We highlighted the adaptative signals associated with a rare genetic disorder of bile acid metabolism and presented their allele frequency trajectories and haplotype networks, which provided a classic example of the evolutionary trade-off between health and fitness.

### Demographic history and genetic structure

Previous studies based on mitochondrial genomes indicated that Shandong served as a cultural crossroads facilitating the migration of ancient individuals from North to South China. Haplogroup analysis also revealed continuous maternal genetic stability in this region from the Early Neolithic to the Late Neolithic Period [38]. Ancient Shandong individuals and ancient coastal southern East Asians were separated into two clusters of approximately eight kya [13]. As an ethnic group dominated by an agricultural civilization, the primary scope of activities for most proto-Han populations remained relatively fixed. However, the Han people have also experienced large-scale or small-scale population migration events for various reasons during prehistoric and historical periods. We integrated ancient and modern genomic datasets and data from 264 newly collected individuals in Shandong Province. Finally, our study leveraged three merged databases, namely, Affy_HO, Affy_HGDP, and Affy_1240K, for detailed genetic analysis. The PCA results revealed a North‒South genetic cline of East Asians, with SDH displaying close genetic affinities with the central Han, Mongolians in Inner Mongolia, Japanese and Koreans (Fig. 1a). However, SDH exhibited a relatively distant genetic relationship with the AA and HM populations. Combined with ancient genomic data, SDH overlapped with ancient YRB-related individuals while displaying a remote genetic relationship with ancient individuals in southern China. The findings revealed via the *F*_ST_, TreeMix, and IBD supported these results. The fineSTRUCTURE and comparison of southern ancestral components further revealed geography/language-related population stratification (Fig. 1c). We also explored the admixture landscape of SDH and identified possible ancestral source candidates based on ADMIXTURE and admixture-*f*_*3*_ statistics, in which ANEA and ASEA contributed unevenly to the gene pool of SDH (Fig. 2a and Supplementary Fig. 2a-b).

SDH is believed to have originated from a YRB-related lineage. Based on the examination of genetic continuity, we still found slight differences between the Yangshao people and the early Neolithic individuals in Shandong (Bianbian, Xiaojingshan, Xiaogao, and Boshan). From the Early to Middle Neolithic, the Siberian-related component declined in Shandong ancients. We detected gene flow between the northern and southern coastal areas. The expansion of the rice farming civilization to the north gradually influenced the Neolithic Longshan culture. There was no significant gene flow from other geographically different ancient people from the late Neolithic to the late Bronze/Iron Age. Additionally, we did not identify other ANEA populations contributing to the SDH gene pool compared with the YR_LBIA population (Supplementary Fig. 6a-c). The qpWave results validated the genetic homogeneity between YR_LBIA and SDH (Fig. 2d). Historically, the migration of SDH toward the South or North was influenced by events such as wars, floods, and other disasters. An example of such migration is the emigration to northeast China, known as the Chuangguandong migration event. However, compared to other Han populations and minority ethnic groups in the YRB, large-scale population migration into Shandong was relatively limited. Therefore, SDH and local temporally diverse ancient people exhibited relative genetic continuity over an extended period. The relative genetic stability can aid in exploring their genetic origin and determining their population history. Additionally, the connection between SDH and East Asian ancestries also underscores the complexity of ancient population dynamics, showing that the history of human migration is characterized by multiple waves of migration, admixture, and genetic exchange.

### Natural selection signals and East Asian-specific variants

Genetic findings from the Chinese Academy of Sciences Precision Medicine Initiative (CASPMI) cohort revealed SNPs associated with waist circumference, BMI, lipid metabolism, and other traits in the northern Han population [68]. Our study also identified some new adaptive signatures associated with BMI-adjusted waist‒hip ratio and height based on the iHS method, implying differences in physique between northern and southern populations (Supplementary Table 13). The PBS results showed that *ABCC11* was under natural selection in the SDH and was associated with the AO and earwax types. The rs17822931 mutation was detected in both dry-type earwax and reduced body odor. We found that the derived allele of rs17822931 was mainly distributed in high-latitude regions and first appeared in Russia approximately 44,000 years ago. The frequency of the T allele has gradually increased in East Asians over the last ten thousand years, and the allele frequency even reached 0.8862, which is significantly different from that in populations on other continents (Supplementary Fig. 9e). As a high-frequency variant specific to East Asians, we need to pay more attention to the effect of *ABCC11.* Previous studies have indicated that this polymorphism could influence estrogen receptor-positive breast cancer, and the T allele might lead to low estrogen efflux activity and increase the risk of breast cancer [69]. From the evolutionary trajectory, we can gain insights into the biological adaptability of geographically distinct populations to their environments and infer population migration and admixture events. The derived allele was mainly distributed in northern populations two thousand years ago (Fig. 4a-c). The T allele frequency increased significantly in southern populations afterward, possibly associated with southward migration.

The *SLC10A1* gene involved in bile acid metabolism was also identified under natural selection in East Asians, but the detailed evolutionary processes and adaptive mechanisms involved remain unknown. The mutation is associated with glycocholic acid, low-density lipoprotein cholesterol, total cholesterol, and uric acid, reflecting *SLC10A1* gene pleiotropism. We explored the evolutionary history of *SLC10A1* from the perspective of allele frequency and a haplotype network. The mutation first appeared in Africans and spread northward and eastward. Arising in the Middle East and Southeast Asia between 2000 and 4000 years ago, the mutation frequency has gradually increased in Asians, with a prominent distribution in southern Chinese people. *SLC10A1* encodes NTCP, and the mutation S267F can decrease the risk of cirrhosis and hepatocellular carcinoma and confer a protective effect against chronic hepatitis B. People carrying S267F exhibit significantly elevated bile acidemia during childhood. In China, we found that northern populations are less likely to suffer from NTCP deficiency disease than southern populations (Fig. 3i). This phenomenon is consistent with the lower prevalence of hepatitis B in northern populations [70]. This regional differentiation may have been shaped by admixture events between southern Chinese populations and Oceanians or by exposure to endemic pathogens (Fig. 4e and Supplementary Fig. 8m). Furthermore, our analysis revealed other natural selection signals associated with metabolism, including *FADS* genes involved in lipid metabolism, *SLC35F3* genes associated with vitamin metabolism, and *ALDH2* genes implicated in alcohol metabolism. These metabolic differences may be due to differences in geographical environments, dietary habits, or exposure to pathogens. Throughout human evolutionary history, multiple factors have acted as driving forces of natural selection. When genotypes are mismatched with the modern environment, it might lead to the manifestation of human diseases. Dissecting the genetic basis of human adaptation in different backgrounds is vital for analyzing genetic diseases.

## Conclusions

Our study elucidated the genetic affinities of Han populations across various regions and examined their interactions with ethnolinguistically diverse neighbors. Genomic data from the SDH population were compared with those from other Han populations in the lower YRB, including Shanxi, Shaanxi, and Henan populations. This comparison revealed a greater degree of genetic continuity in Shandong populations from the late Bronze/Iron Age to the present. Furthermore, numerous natural selection signals were identified, clarifying the evolutionary trajectory of East Asian-specific traits related to axillary odor and bile acid metabolism. The *ABCC11* gene variant rs17822931, which may be associated with environmental adaptations, was detected approximately 4,400 years ago. The earliest occurrence of the *SLC10A1* gene variant (rs2296651) was identified approximately 10,000 years ago and is linked to NTCP deficiency and chronic hepatitis B. These findings provide compelling genetic evidence for understanding the origin and progression of East Asian-specific traits and diseases.

## Methods

### Sample collection and DNA preparation

We collected saliva samples from 264 unrelated healthy Han individuals in Shandong Province, North China. A QIAamp DNA Mini Kit (QIAGEN, Germany) was used to extract and purify the DNA. Subsequently, a quantitative analysis was conducted using the Qubit dsDNA HS Assay Kit from Thermo Fisher Scientific on a Qubit 3.0 fluorometer following the protocols provided by the manufacturer. The Medical Ethics Committees of West China Hospital of Sichuan University reviewed and approved the project and corresponding protocols. The individuals participating in this study were randomly selected, and informed consent was obtained from all participants. All included individuals were required to be indigenous residents with at least three generations at the sampling sites. All procedures were performed following the Helsinki Declaration of 2013 [71].

### Quality control, genotype calling, and dataset merging

The Affymetrix Array was used for genotyping 264 individuals first reported here. We used PLINK v.1.90 [72] and King [73] to assess genetic relatedness between all pairs of samples. Individuals whose relatives were present within the three generations were removed. The following parameters were used to filter SNPs and samples (mind: 0.05, geno: 0.05, and HWE: 10^–6^. Approximately 465 K SNPs passed the first filtering step. We merged the database with publicly available and previously published data generated via an Affymetrix chip. The detailed populations and their classification information from the Affymetrix dataset are shown in Supplementary Table 17. Our data (including 2808 individuals from different language families) were merged with HGDP [74] and Oceania genomic resources [75] to form a global high-density dataset (424,501 SNPs). Then, variants from the Affymetrix dataset and the Allen Ancient DNA Resource (HO dataset and 1240 K dataset, https://reich.hms.harvard.edu/datasets) were merged to generate the middle-density dataset (including 359,009 SNPs) and low-density dataset (including 119,114 SNPs). Finally, three merged datasets (Affy_HGDP, Affy_1240K, and Affy_HO) were generated.

### Principal component analysis

PCA was conducted using the smartpca package in EIGENSOFT, which focuses on modern and ancient populations at the East Asian and Chinese levels. The analysis used the following parameters: numoutlieriter: 0 and lsqproject: YES. East Asian-scale PCA was conducted to explore the population structure between the newly studied population and ancient/modern East Asian populations based on the HO_Affy database. To further dissect the fine-scale genetic structure of populations from the lower YRB, we also performed Chinese-scale PCA to dissect the genetic relationship between the target population and ANEA, in which modern people were projected onto the context of ancient individuals.

### *FST* calculation and TreeMix

To quantify the genetic distance precisely, we used PLINK v.1.90 to calculate the pairwise fixation index (*F*_ST_) between the target and reference populations. We also applied TreeMix v.1.1365 based on allele frequencies to explore their phylogenetic relationships. The number of migration edges was set from 0 to 7. A maximum likelihood tree was constructed by replication for each migration number.

### Haplotype-based population analysis

We implemented SHAPEIT v.2.0 [76] to estimate the haplotypes based on the HGDP_Affy database and applied the recommended genetic map [77]. We used the default parameters (–burn 10 –prune 10 –main 30) [76]. Subsequently, we detected the pairwise shared IBD segments with Refined-IBD software and calculated the IBD matrix among pairwise populations based on the different IBD lengths. The observed IBD blocks with different lengths indicate differentiated genetic interactions occurring at different time horizons. To determine the recent demographic history of people with SDH, we estimated the effective population size within 150 generations using IBDNe v23Apr20 [78]. We used ChromoPainter and fineSTRUCTURE v4 [79] to estimate the fine-scale population structure among Han Chinese people and geographical neighbors based on the coancestry matrix, which revealed ancestral relationships at the individual level. We randomly selected certain individuals from 24 populations to assess phylogenetic relationships using fineSTRUCTURE, ChromoCombine, and ChromoPainter [80] with the following parameters: -s3iters 100,000, -s4iters 50,000, -s1 minsnps 1000, and s1indfrac 0.1. We also calculated the ROH in the SDH population and ethnolinguistically different populations. Additionally, we used PLINK v.1.90 to classify ROH with lengths of < 1, 1–5, and > 5.

### Admixture analysis

We used unsupervised model-based ADMIXTURE analyses to estimate the ancestry proportion of each individual. The genomic data were LD-pruned using PLINK v.1.9 with these parameters (–indep-pairwise 200 25 0.4). We assumed that the number of predefined ancestral sources ranged from 2 to 20. The models with K = 6 and 3 and the lowest cross-validation error were chosen as the best-fit models among our major admixture models.

### F-statistics

We calculated admixture-*f*_3_ statistics to test potential admixture signals of SDH using qp3Pop in ADMIXTOOLS [81]. If* f*_3_-values in the form of *f*_3_ (Reference, Reference; SDH) were negative with Z < -3, it indicated that two reference populations were predefined ancestral surrogates of SDH. In addition, *f*_4_ statistics were calculated using qpDstat implemented in ADMIXTOOLS. We then used different forms of *f*_4_ statistics to explore genetic affinities and differentiated gene flow events between the target and other ancient/modern reference populations.

### QpAdm and qpWave

We modeled the ancestry admixture composition of representative ancient ancestral sources of SDH using two-way qpAdm modeling. We used the default parameter settings: allsnps: YES; details: YES. Moreover, a series of principles were used to screen the best-fitting qpAdm [82] models. First, the ancestry portion was greater than 0 and less than 1. Second, the standard error is smaller than the estimated minimum values of ancestry admixture proportion. Third, each p-value of the tested admixture model was greater than 0.05. We used Mbuti, Iran_GanjDareh_N, Italy_North_Villabruna_HG, Ami, Mixe, Onge, Papuan, China_Tianyuan, Ust_Ishim and Australia as outgroups. Two-way qpAdm admixture models can be used to successfully elucidate the gene pool of SDH. Using the same outgroup sets, we also implemented the qpWave package in ADMIXTOOLS to test genetic homogeneity among Han Chinese and other ancient and modern populations. The p values of pairwise populations are presented in the form of a heatmap.

### ALDER

Admixture times with different sources were estimated via admixture-induced linkage disequilibrium for evolutionary relationships implemented in ALDER v1.03 [83]. The parameters used were as follows: mindis, 0.005; jackknife, YES.

## Identification of natural selection signals

First, we estimated HDVs between northern and southern Han Chinese populations based on allele frequency (*F*_ST_). Subsequently, we used PBS to explore natural selection signals in the target population. The formula is as follows: PBS_A_ = (T_AB_ + T_AC_—T_BC_)/2, T = -lg(1-*F*_ST_) [84]. We defined A as the studied population, while B and C were the ingroup and outgroup, respectively. We conducted different scales of PBS focused on genes with the top 0.1% of PBS values. In the distant test models, we used Europeans as the outgroup and Hlai as the ingroup. We used Hlai as the outgroup and Han people from Hunan Province as the ingroup to test the population-specific selection signatures. We further validated biological adaptive variants by iHS and XP-EHH using selscan v1.2.0 [85], and HNL was selected as the reference population. The PBS values of the variation need to be in the top 0.1%, which can be verified by one of the *F*_ST_ and iHS/XP-EHH methods. Finally, we obtained a high-quality set of selected alleles and used VEP to annotate them. We annotated trait-related selection candidate variants in the GWAS catalog. We used Metascape to perform GO and KEGG enrichment analyses [86]. We constructed linkage disequilibrium plots using Haploview [87]. The haplotype networks were constructed by PopART [88] using the 11 SNPs in the *SLC10A1* gene and 6 SNPs in the *ABCC11* gene from Affy_HGDP.

### Supplementary Information

Supplementary Material 1.

Supplementary Material 2.

## Data Availability

The raw data derived from human samples have been deposited in the Zenodo ( https://zenodo.org/records/11549865) with accession number 11549865 and OMIX database (https://ngdc.cncb.ac.cn/omix/release/OMIX005781) with the accession number OMIX005781. Reference genotype data for ancient and modern individuals were collected from the Allen Ancient DNA Resource (https://reich.hms.harvard.edu/allen-ancient-dna-resource-aadr-downloadable-genotypes-present-day-and-ancient-dna-data). The access and use of the data complied with the regulations of the People's Republic of China on the administration of human genetic resources.
