# Supplementary material for: Population genetic admixture and evolutionary history in the Shandong Peninsula inferred from integrative modern and ancient genomic resources
Source: BMC Genomics. 2024 Jun 18;25:611. doi: 10.1186/s12864-024-10514-9 (PMC11184692; doi:10.1186/s12864-024-10514-9)
Supplement: Supplementary file 1 — Supplementary Material 1. [file 12864_2024_10514_MOESM1_ESM.docx]

**Supplemental Figures** for

**Genetic admixture and evolutionary history of Han Chinese in the Shandong Peninsula inferred from integrative modern and ancient genomic resource**

Haoran Su^1,2,3^^†^, Mengge Wang^2,4,5†,*^, Xiangping Li^2,6^, Shuhan Duan^2,5,7^, Qiuxia Sun^2,8^, Yuntao Sun^2,9^, Zhiyong Wang^2,6^, Qingxin Yang^2,6^, Yuguo Huang^2^, Jie Zhong^2^, Jing Chen^2,10^, Xiucheng Jiang^2,5,7^, Jinyue Ma^2,5,7^, Ting Yang^2,6^, Yunhui Liu^2,8^, Lintao Luo^2,8^, Yan Liu^2,5,7^, Junbao Yang^5,7^, Gang Chen^12^, Chao Liu^11*^, Yan Cai^1†,*^, Guanglin He^2,4,5*^

^1^Genetic and Prenatal Diagnosis Center, Affiliated Hospital of North Sichuan Medical College, Nanchong, Sichuan, 637007, China. ^2^Institute of Rare Diseases, West China Hospital of Sichuan University, Sichuan University, Chengdu, 610000, China. ^3^School of Laboratory Medicine, North Sichuan Medical College, Nanchong, Sichuan, 637007, China. ^4^Center for Archaeological Science, Sichuan University, Chengdu, 610000, China. ^5^Research Center for Genomic Medicine, North Sichuan Medical College, Nanchong, 637100, China. ^6^School of Forensic Medicine, Kunming Medical University, Kunming, 650500, China. ^7^Institute of Basic Medicine and Forensic Medicine, North Sichuan Medical College and Genetic and Prenatal Diagnosis Center, Affiliated Hospital of North Sichuan Medical College, Nanchong, Sichuan, 637007, China. ^8^Department of Forensic Medicine, College of Basic Medicine, Chongqing Medical University, Chongqing, 400331, China. ^9^West China School of Basic Science & Forensic Medicine, Sichuan University, Chengdu, 610041, China. ^10^School of Forensic Medicine, Shanxi Medical University, Jinzhong, 030001, China. ^11^Anti-Drug Technology Center of Guangdong Province, Guangzhou, 510230, China. ^12^Hunan Key Laboratory of Bioinformatics, School of Computer Science and Engineering, Central South University, Changsha 410075, China.

^†^Haoran Su, Mengge Wang and Yan Cai contributed equally to this work.

Correspondence: Mengge Wang ([Menggewang2021@163.com](mailto:Menggewang2021@163.com)), Chao Liu ([liuchaogzf@163.com](mailto:liuchaogzf@163.com)), Yan Cai ([caiyandd@163.com](mailto:caiyandd@163.com)), Guanglin He ([guanglinhescu@163.com](mailto:guanglinhescu@163.com)).

**Contents**

**Supplemental Figures**

**Figure S1**	3

**Figure S2**	4

**Figure S3**	5

**Figure S4**	6

**Figure S5**	7

**Figure S6**	8

**Figure S7**	9

**Figure S8**	10

**Figure S9**	11

**Figure S1. Sampling distribution and genetic affinities of SDH.**

(**a**) Geographical position of Shandong province in China. The orange dots in this map show the selected locations from which Shandong individuals were sampled for this study. (**b-c**) An inferred TreeMix-based phylogenetic relationship with the optimal number of migration edges being three. (**d**) Shared identity-by-descent (IBD) model of total length among ethnolinguistically diverse East Asia populations. (**e**) Runs of homozygosity among 24 East Asia populations. (**f**) ADMIXTURE analysis of geographically different populations based on the high-density Affy dataset.

**Figure S2. Admixture scenarios of SDH inferred from the *f*_3_-statistics.**

(**a**) Admixture-*f*_3_ heatmap using plausible ancestral sources based on the merged 1240K_Affy dataset, with admixture signatures indicated by the symbol “+”. (**b**) Admixture-*f*_3_ using modern populations as plausible ancestral sources based on the merged HGDP_Affy dataset.

**Figure S3. Asymmetric *f*_4_ tests of SDH.**

(**a-b**) The *f*_4_-statistics in the form of *f*_4_(ANSE, SDH; Reference, Mubti), where ANSE represented China_NEastAsia_Coastal_EN and China_YR_MN, respectively. Reference represented global populations based on the 1240K_Affy dataset. Significant negative values indicated SDH had additional gene flow events compared with ANEA. (**c**) *F*_4_ tests in the form of *f*_4_(ASEA, SDH; Reference, Mubti), where ASEA represented Taiwan_Hanben_IA.

**Figure S4. Asymmetric *f*_4_ analysis based on the merged HGDP_Affy.**

(**a-c**) *F*_4_-statistics in the form of *f*_4_(southern Han, SDH; Reference, Mubti), where Chongqing Han, Hunan Han, and Yunnan Han were chosen as the representatives of southern Han.

**Figure S5. The genetic location and effective population size of Han in the lower YRB.**

(**a**) Modern populations were projected onto ancient individuals, and PCA showed the clustering patterns between Han populations in the YRB and ancient YRB-related individuals. (**b**) The effective population size of four Han populations from Shaanxi, Shandong, Henan, and Shanxi.

**Figure S6. Three *f*_4_-statistics tests were calculated to explore the genetic continuity in the YRB from the Early Neolithic to the Late Bronze/Iron Age.**

**(a)** *F*_4_-statistics test in the form of *f*_4_(China_NEastAsia_Coastal_EN, China_YR_MN; Reference, Mbuti). (**b**) *F*_4_-statistics test in the form of *f*_4_(China_YR_MN, China_YR_LN; Reference, Mbuti). (**c**) *F*_4_-statistics test in the form of *f*_4_(China_YR_LN, China_YR_LBIA; Reference, Mbuti).

**Figure S7. The genetic stability of four Han populations in the YRB is inferred from the asymmetric *f*_4_-statistics.**

(**a-d**) *F*_4_-statistics test in the form of *f*_4_(China_YR_LBIA，Henan/Shaanxi/Shandong/Shanxi Han; Reference, Mbuti) based on the merged 1240K_Affy dataset.

**Figure S8. Natural selection and evolutionary trajectories of *ABCC11* and *SLC10A1*.**

(**a**) The Manhattan plot showed the biological adaptative signals inferred from PBS in the SDH-HNH-HNL trio model. (**b**) Sankey diagram reflected the results of genome-wide Associations using the GWAS catalog. (**c**) The enrichment analysis among adaptative genes. (**d-g**) Allele frequencies of highly differentiated variants located in *FADS2*, *FADS1*, *LILRA3*, *CR1* genes. (**h-j**) The evolutionary trajectory of rs17822931 is located in the *ABCC11* gene from 10,000 to 2000 years ago. (**k-m**) Evolutionary trajectory of rs2296651 located in the *SLC10A1* gene from 10,000 to 2000 years ago.

**Figure S9. Linkage disequilibrium, haplotype frequencies and allele frequency trajectories.**

(**a**) A linkage disequilibrium plot was constructed from 17 SNPs located in *ABCC11*. (**b**) The pattern of linkage disequilibrium in *SLC10A1* from bp 70238964 to bp 70261665. (**c**) The haplotype frequencies of the *ABCC11* gene*.* (**d**) The haplotype frequencies of *SLC10A1.* (**e**): The allele frequency evolutionary trajectories. (**f**) Extended haplotype homozygosity of rs2296651 located in *SLC10A1* gene. (**g-h**) Allele frequencies of rs72474224 and rs2274084 located in *GJB2* gene.
